# Association Between Household Type and Cardiovascular Disease Prevalence: A Korean National Health and Nutrition Examination Survey Study (2019–2021)

**DOI:** 10.3390/healthcare14213757

**Published:** 2026-11-04

**Authors:** Hyun-Su Kim

**Affiliations:** Department of Nursing, Kyungdong University, 815 Gyeonhwon-ro, Munmak, Wonju 26495, Gangwon-do, Republic of Korea; elegan80@sangji.ac.kr; Tel.: +82-33-738-1439; Fax: +82-33-738-1449

**Keywords:** one-person household, disease risk, hypertension, dyslipidemia, ischemic heart disease, national study

## Abstract

Background/Objectives: To investigate the association between household type and the prevalence of hypertension, dyslipidemia, and ischemic heart disease, with a focus on the implications for cardiovascular disease prevention and management in one-person households. Methods: This study used data from 15,913 adults aged ≥19 years from the eighth Korea National Health and Nutrition Examination Survey conducted once per year between 2019 and 2021. Results: Significant differences in demographic characteristics, socioeconomic status, comorbidities, and health-related behaviors were observed between one-person and multi-person households. In unadjusted analyses, living alone was associated with higher odds of hypertension, dyslipidemia, and ischemic heart disease. After adjustment for age, sex, socioeconomic characteristics, health-related behaviors, and comorbidities, living alone remained significantly associated with hypertension (OR = 1.149, 95% CI: 1.022–1.292), whereas the associations with dyslipidemia (OR = 1.007, 95% CI: 0.893–1.136) and ischemic heart disease (OR = 1.143, 95% CI: 0.901–1.451) were not statistically significant. Conclusions: Living alone was associated with a higher prevalence of hypertension after adjustment for demographic, socioeconomic, behavioral, and comorbidity-related factors. In contrast, the initially observed associations with dyslipidemia and ischemic heart disease were attenuated after adjustment. These findings suggest that differences in individual characteristics may account for a substantial portion of the crude associations between household type and cardiovascular conditions. Given the cross-sectional design, causal or temporal relationships cannot be established.

## 1. Introduction

Cardiovascular diseases are disorders affecting the heart and blood vessels, including ischemic heart diseases such as angina pectoris and myocardial infarction. Hypertension and dyslipidemia are major cardiovascular risk factors that contribute to the development of cardiovascular diseases [1]. Cardiovascular disease is one of the most common causes of death worldwide [2]; according to the American Heart Association, there were 19.055 million deaths due to cardiovascular disease worldwide in 2020, which increased by 18.71% from 2010 [3]. Moreover, the number of countries with gradually decreasing mortality rates related to cardiac disease is increasing [4]. Hence, a vital issue for this group of diseases is the substantially increasing prevalence compared with the decreasing trend in mortality rate [5]. Cardiovascular disease is becoming a chronic condition and thus a burden on mental health, physical health, and the economy via increasing medical expenditures during disease management and treatment [2,3]. The factors associated with cardiovascular health can be divided into two main categories [6]: (1) health status; the prevalence rate increases if there is an underlying disease such as hypertension, hyperlipidemia, or diabetes mellitus [7]; and (2) health behaviors such as diet, exercise, and smoking. Many studies have reported that cardiovascular disease is closely related to an individual’s daily lifestyle habits and health status [6,8,9].

A meta-analysis on the association between one-person households and cardiovascular disease reported that people living alone may have an increased risk of cardiovascular disease [10]. The increase in the number of one-person households is a phenomenon that is rapidly occurring worldwide [11], reflecting socio-demographic changes such as increased life expectancy, increased late marriage, avoidance of marriage, increased divorce rate, separation of families due to the socio-economic attraction structure in urban areas, as well as increased personal autonomy and independence [12]. One-person households are known to have poorer household incomes, expenditures, living environments, nutritional intakes, and health responses than those that are multi-person [13], and the combined effects of these factors make it difficult to maintain and improve healthy daily lifestyle habits [14].

The environmental characteristics of one-person households lead to insufficient healthcare, resulting in increased chronic diseases and overall social problems such as increased medical expenses, decreased social activities due to disease, and increased economic burden due to decreased income [15].

This study aimed to examine the association between household type and the prevalence of hypertension, dyslipidemia, and ischemic heart disease among Korean adults using data from the 2019–2021 Korea National Health and Nutrition Examination Survey (KNHANES). The findings may provide evidence to inform future cardiovascular disease prevention and management strategies for one-person households.

## 2. Materials and Methods

### 2.1. Study Design and Population

The KNHANES has been conducted every year, based on Article 16 of the National Health Promotion Act, to identify the health and nutrition state of Koreans since 1998 by the Korea Disease Control and Prevention Agency. This is a statutory survey conducted by trained investigators visiting households selected as samples and conducting one-on-one interviews using a survey program. Data are collected by classifying them into household member confirmation, health questionnaire, examination, and nutrition surveys, and the examination survey includes physical measurements, blood tests, urinalysis, oral examinations, pulmonary function tests, and ophthalmic examinations. This nationally approved statistical data was provided after approval for data use in accordance with the raw data request procedures and regulations.

This study analyzed the raw data of the eighth KNHANES conducted once per year for 3 years between 2019 and 2021. A total of 22,559 adults participated, including 8110 in 2019, 7359 in 2020, and 7090 in 2021. Excluding participants with any missing variables, a final analysis was conducted with data from 15,913 adults aged ≥19 years.

### 2.2. Measures

#### 2.2.1. Type of Household

Household type was classified based on the survey results on the number of household members. If the respondent answered that the number of household members was one, it was classified as a one-person family, and if the respondent answered that other people were living with them, it was classified as a multi-person family, regardless of the number of members.

#### 2.2.2. Cardiovascular Conditions and Risk Factors

The study outcomes included hypertension and dyslipidemia as cardiovascular risk factors and ischemic heart disease, including myocardial infarction and angina pectoris, as a cardiovascular disease. Each condition was defined by an answer of “yes” to the corresponding item asking whether the respondent had been diagnosed with hypertension, dyslipidemia, myocardial infarction, or angina pectoris by a doctor.

#### 2.2.3. General Characteristics

The overall participant characteristics included demographics, socioeconomics, disease conditions, lifestyle habits, and health status, and demographic characteristics included sex, age, and marital status, including cohabitation status. For socioeconomic characteristics, education level, occupation type, and personal income level were analyzed. Disease conditions were analyzed for hypertension, dyslipidemia, stroke, ischemic heart disease, osteoarthritis, rheumatoid arthritis, osteoporosis, diabetes mellitus, renal failure, and depression. As for lifestyle habits and health status characteristics, stress level, smoking, drinking, body mass index (BMI), weight management status, weekly aerobic physical activity practice, systolic and diastolic blood pressure, total cholesterol, fasting blood glucose, and glycated hemoglobin levels were analyzed.

Sex was classified as male and female, and age was classified as <45 years, 45–54 years, 55–64 years, and ≥65 years. Marital status was classified using cohabitation, separated or widowed, divorced, and never married. Additionally, if a person was married and had a spouse but lived separately for personal reasons, such as in the case of a weekend couple, they were classified as a one-person household even though they were married (with spouse). Education level was classified and analyzed as elementary school graduate or lower, middle school graduate, high school graduate, and university graduate or higher. Occupation types were classified as managers/professionals/office workers, service and sales workers, agricultural and fishery workers, technicians/machine operators/simple labor workers, military personnel, and unemployed. Personal income level was classified as low, medium-low, medium-high, and high. Stroke, osteoarthritis, rheumatoid arthritis, osteoporosis, diabetes mellitus, renal failure, and depression were analyzed based on the survey results, depending on whether a doctor diagnosed each disease. Regarding lifestyle habits and health status characteristics, stress level was classified as feeling an extreme amount, feeling a lot, feeling a little, or hardly feeling. Smoking was classified according to current smoking status as smoking every day, smoking occasionally, smoking in the past but not currently, or never smoking. Drinking was reclassified into no drinking, about once a month, about two to four times a month, and more than twice a week, based on the item regarding drinking frequency during the past year. Weight-management status was classified into no management, effort to lose, effort to maintain, and effort to gain. For physical activity practice, whether the person engaged in moderate-intensity physical activity for more than 2.5 h per week, high-intensity physical activity for more than 1.25 h per week, or a combination of moderate-intensity and high-intensity physical activity (1 min of high intensity was 2 min of moderate-intensity) for the equivalent amount of time for each activity was analyzed. After measuring systolic and diastolic blood pressures three times directly, the variables for the mean blood pressure values were analyzed. Total cholesterol, fasting blood glucose, and glycated hemoglobin levels were analyzed based on measurement data obtained after blood tests.

### 2.3. Statistical Analysis

The collected data were analyzed using SAS version 9.4 (SAS Institute Inc., Cary, NC, USA), and statistical significance was set at a two-sided *p*-value < 0.05.

Continuous variables are presented as means and standard deviations, and categorical variables as frequencies and percentages. Differences between one-person and multi-person households were evaluated using an independent *t*-test or Wilcoxon rank-sum test for continuous variables and a chi-square test or Fisher’s exact test for categorical variables, as appropriate. Logistic regression analyses were performed to examine the associations between household type and the prevalence of hypertension, dyslipidemia, and ischemic heart disease. Multi-person households were used as the reference group. Three models were constructed for each outcome. Model 1 was unadjusted. Model 2 was adjusted for age and sex. Model 3 was additionally adjusted for education level, personal income level, smoking, drinking, aerobic physical activity, diabetes mellitus, and depression. The results are presented as odds ratios (ORs) with 95% confidence intervals (CIs).

## 3. Results

### 3.1. Characteristics of Participants

Table 1 shows differences in general characteristics according to the type of household. In terms of demographic characteristics, significant differences were found in sex (*p* = 0.002), age (*p* < 0.001), and marital status (*p* < 0.001). Regarding socioeconomic characteristics, significant differences were identified in education level *p* < 0.001), occupational type (*p* < 0.001), and personal income level (*p* < 0.001). Regarding marital status, there were 259 one-person households where participants had a spouse but lived alone, as a weekend couple, or in other forms due to personal circumstances, accounting for 11.77% of all one-person households. In terms of disease conditions, there were significant differences in the prevalence of hypertension (*p* < 0.001), dyslipidemia (*p* < 0.001), stroke (*p* < 0.001), ischemic heart disease (*p* < 0.001), osteoarthritis (*p* < 0.001), rheumatoid arthritis (*p* = 0.001), osteoporosis (*p* < 0.001), diabetes mellitus (*p* < 0.001), renal failure (*p* = 0.003), and depression (*p* < 0.001). Exploring lifestyle habits and health status characteristics, statistically significant differences were found in stress levels (*p* < 0.001), smoking (*p* < 0.001), drinking (*p* < 0.001), weight-management status (*p* < 0.001), aerobic physical activity (*p* < 0.001), systolic blood pressure (*p* < 0.001), total cholesterol (*p* < 0.001), fasting blood glucose (*p* < 0.001), and glycated hemoglobin levels (*p* < 0.001). However, BMI (*p* = 0.450) and diastolic blood pressure (*p* = 0.150) did not show significant differences.

### 3.2. Cardiovascular Disease According to the Type of Household

Table 2 presents the associations between household type and the prevalence of hypertension, dyslipidemia, and ischemic heart disease. In the unadjusted model (Model I), individuals living alone had significantly higher odds of hypertension (OR = 1.818, 95% CI: 1.652–2.000), dyslipidemia (OR = 1.461, 95% CI: 1.319–1.618), and ischemic heart disease (OR = 1.701, 95% CI: 1.368–2.116) than those living in multi-person households. After adjustment for age and sex, the associations were substantially attenuated and remained statistically significant only for hypertension (OR = 1.234, 95% CI: 1.104–1.378). In the multivariable-adjusted model (Model III), living alone remained significantly associated with hypertension (OR = 1.149, 95% CI: 1.022–1.292, *p* = 0.020), whereas the associations with dyslipidemia (OR = 1.007, 95% CI: 0.893–1.136, *p* = 0.905) and ischemic heart disease (OR = 1.143, 95% CI: 0.901–1.451, *p* = 0.270) were not statistically significant.

## 4. Discussion

The principal finding of this study was that the associations between household type and cardiovascular conditions differed substantially after adjustment for potential confounding factors. Although individuals living alone had higher crude odds of hypertension, dyslipidemia, and ischemic heart disease, adjustment for age and sex markedly attenuated these associations. After further adjustment for socioeconomic characteristics, health-related behaviors, and comorbidities, living alone remained significantly associated only with hypertension. In contrast, the associations with dyslipidemia and ischemic heart disease were no longer statistically significant. These findings suggest that demographic, socioeconomic, behavioral, and health-related differences between household types may account for a substantial proportion of the crude associations observed for dyslipidemia and ischemic heart disease. However, given the cross-sectional nature of this study, these associations should not be interpreted as evidence that living alone directly contributes to the development of cardiovascular disease. This finding aligns with previous studies that reported that one-person households have decreased health levels and major health indicators compared with multi-person households [15,16,17]. Furthermore, a previous study analyzing individual lifestyle habits as factors related to cardiovascular disease risk showed that the risk of developing cardiovascular disease decreases as sleep time increases and drinking habits improve [15]. In addition, according to a gender comparison study on the development of cardiovascular disease, education and income levels were common predictive factors of the risk of developing cardiovascular disease for both men and women [17]. Previous research has identified associations between socioeconomic status, health-related behaviors, and access to healthcare services [18]. Furthermore, a large prospective cohort study demonstrated that lifestyle factors partially mediated the association between socioeconomic status and cardiovascular outcomes [19]. In this study, significant differences in education level, occupation type, and personal income level were observed between one-person and multi-person households. Most people were classified as having low education and income levels, with many simple labor workers and unemployed people in one-person households. Socioeconomic status affects the health-related environment by being involved in biological factors, behavioral characteristics, and physical and mental health [18]. In particular, an association has been reported between the risk of developing cardiovascular disease and income level, education level, employment status, and neighborhood socioeconomic factors [18]. Hence, the influence of socioeconomic level on chronic disease management is crucial. Furthermore, when planning to establish a reasonable medical support system, it may be necessary to determine the qualitative level of medical service use, in addition to the quantitative aspects among one-person households, to evaluate healthcare ability according to socioeconomic status.

Studies on the health behavior in one-person households have reported that depression is particularly closely related to socioeconomic level and is a factor affecting health risk [15,20,21,22]. In this study, both history of depression diagnosis and stress levels showed significant differences depending on the type of household. This may be because although independence is guaranteed in the life of a one-person household, opportunities to receive emotional support and relieve stress by forming empathetic relationships with family are reduced. In addition, smoking, drinking, and physical activity levels also significantly differed between one-person and multi-person households. Improvement in lifestyle habits reduces obesity [23] and increases self-management, which has a positive effect on the treatment of diseases such as diabetes mellitus, improving an individual’s overall health status [24]. In other words, physical health and stress management are crucial factors for a healthy life. Several explanations should be considered when interpreting the observed associations. Differences in socioeconomic circumstances, psychological well-being, and health-related behaviors may contribute to the relationship between household type and cardiovascular health. However, the direction of this relationship remains uncertain. Individuals with pre-existing cardiovascular conditions or other health problems may experience changes in their household arrangements, suggesting the possibility of reverse causality. Furthermore, age and socioeconomic differences between household types may partly explain the observed associations. Therefore, the present findings should be interpreted as associations rather than evidence of a direct causal effect of living alone on cardiovascular disease.

In terms of sex, in contrast to the present study’s findings, a study by Gandhi et al. [25] showed that living alone was not independently associated with cardiovascular disease-related events; however, significant differences by gender were evident, and men living alone had worse outcomes regarding cardiovascular disease than women. Factors predicting the risk of cardiovascular disease in male members of one-person households were more diverse than in female members of one-person households [17]. Based on this, it can be hypothesized that cardiovascular disease in male members of one-person households is a cumulative problem of gender characteristics, such as relatively inadequate lifestyle management, compared with their female counterparts. However, in this study, the proportion of women in single-person households was higher. Therefore, as various results are reported based on gender, it may be necessary to apply a gender-based approach for continuous disease management in one-person households.

Socioeconomic and health-related characteristics differed substantially between one-person and multi-person households in this study. These differences should be considered when interpreting associations between household type and cardiovascular health.

### Study Limitations and Strengths

This study has several limitations. First, because of its cross-sectional design, the temporal relationship between household type and cardiovascular disease could not be established. Reverse causality is possible, as pre-existing health conditions may influence individuals’ household arrangements. Consequently, the observed associations should not be interpreted as causal relationships. Longitudinal studies are needed to determine whether changes in household type precede or follow the development of cardiovascular conditions.

Second, cardiovascular conditions were identified using self-reported physician diagnoses rather than independently verified medical records or standardized clinical diagnostic assessments. Therefore, recall bias and misclassification cannot be excluded. Furthermore, differences in healthcare utilization between one-person and multi-person households may have influenced the likelihood of receiving a diagnosis. For example, individuals with comorbidities may have more frequent contact with healthcare services, potentially increasing disease detection. Conversely, limited access to healthcare may result in underdiagnosis. These possibilities raise concerns about ascertainment bias, which should be considered when interpreting the observed associations.

Despite these limitations, this study provides information on differences in cardiovascular disease prevalence and health-related characteristics between one-person and multi-person households using a large national survey dataset. However, the exclusion of participants with missing data may have introduced selection bias and may limit the generalizability of the findings. Further longitudinal studies are needed to clarify the temporal relationship between household type and cardiovascular health.

## 5. Conclusions

This study found that living alone was associated with a higher prevalence of hypertension even after adjustment for demographic, socioeconomic, behavioral, and comorbidity-related factors. In contrast, the crude associations between living alone and dyslipidemia and ischemic heart disease were attenuated and were no longer statistically significant after adjustment. These findings indicate that differences in individual characteristics between household types should be considered when examining cardiovascular health disparities. Given the cross-sectional design, causal or temporal relationships cannot be established. Further longitudinal studies are needed to clarify the relationship between household type and cardiovascular health.

## Figures and Tables

**Table 1:** Difference in general participant characteristics by household type (*n* = 15,913).

Characteristics			Family Structure				
	Variable	Category	*n* (%)	One-Person (*n* = 2200)	Multi-Person (*n* = 13,713)	**χ² or t(Z)**	*p*
				*n* (%) or M ± SD [min, max]	*n* (%) or M ± SD [min, max]		
Demographic	Sex	Males	7113 (44.70)	918 (41.73)	6195 (45.18)	9.12	0.002
		Females	8800 (55.30)	1282 (58.27)	7518 (54.82)		
	Age (years)	<45	5587 (35.11)	597 (27.14)	4990 (36.39)	530.07	<0.001
		45–54	2900 (18.22)	214 (9.73)	2686 (19.59)		
		55–64	3261 (20.49)	384 (17.45)	2877 (20.98)		
		≥65	4165 (26.18)	1005 (45.68)	3160 (23.04)		
	Marital status	Spouse, cohabitated	10,621 (66.75)	259 (11.77)	10,362 (75.57)	4631.15	<0.001
		Spouse, separated or widowed	1409 (8.85)	840 (38.18)	569 (4.15)		
		Divorced	799 (5.02)	379 (17.23)	420 (3.06)		
		Never married	3084 (19.38)	722 (32.82)	2362 (17.22)		
Socio- Economic	Education	Elementary school or lower	2771 (17.41)	756 (34.36)	2015 (14.69)	558.21	<0.001
		Middle school	1537 (9.66)	255 (11.59)	1282 (9.35)		
		High school	5454 (34.27)	587 (26.68)	4867 (35.49)		
		College or higher	6151 (38.66)	602 (27.37)	5549 (40.47)		
	Occupation type	Managers/ professionals/office workers	3957 (24.87)	415 (18.86)	3542 (25.83)	92.47	<0.001
		Service and sales workers	2099 (13.19)	247 (11.23)	1852 (13.51)		
		Agricultural and fishery workers	575 (3.61)	58 (2.64)	517 (3.77)		
		Technicians/machine operators/simple labor workers	3067 (19.27)	460 (20.91)	2607 (19.01)		
		Military personnel	23 (0.14)	1 (0.05)	22 (0.16)		
		Unemployed	6192 (38.92)	1019 (46.31)	5173 (37.72)		
	Personal income level	Low	3852 (24.20)	922 (41.91)	2930 (21.37)	545.07	<0.001
		Medium-low	3949 (24.82)	580 (26.36)	3369 (24.57)		
		Medium-high	4067 (25.56)	430 (19.55)	3637 (26.52)		
		High	4045 (25.42)	268 (12.18)	3777 (27.54)		
Disease	Hypertension	Yes	4027 (25.31)	791 (35.95)	3236 (23.60)	153.14	<0.001
		No	11,886 (74.69)	1409 (64.05)	10,477 (76.40)		
	Dyslipidemia	Yes	3450 (21.68)	608 (27.64)	2842 (20.72)	53.33	<0.001
		No	12,463 (78.32)	1592 (72.36)	10,871 (79.28)		
	Stroke	Yes	346 (2.17)	89 (4.05)	257 (1.87)	42.02	<0.001
		No	15,567 (97.83)	2111 (95.95)	13,456 (98.13)		
	Ischemic heart disease	Yes	507 (3.19)	107 (4.86)	400 (2.92)	23.29	<0.001
		No	15,406 (96.81)	2093 (95.14)	13,313 (97.08)		
	Osteoarthritis	Yes	1844 (11.59)	445 (20.23)	1399 (10.20)	185.98	<0.001
		No	14,069 (88.41)	1755 (79.77)	12,314 (89.80)		
	Rheumatoid arthritis	Yes	302 (1.90)	62 (2.82)	240 (1.75)	11.62	0.001
		No	15,611 (98.10)	2138 (97.18)	13,473 (98.25)		
	Osteoporosis	Yes	1289 (8.10)	367 (16.68)	922 (6.72)	252.55	<0.001
		No	14,624 (91.90)	1833 (83.32)	12,791 (93.28)		
	Diabetes mellitus	Yes	1673 (10.51)	326 (14.82)	1347 (9.82)	50.29	<0.001
		No	14,240 (89.49)	1874 (85.18)	12,366 (90.18)		
	Renal failure	Yes	176 (1.11)	38 (1.73)	138 (1.01)	9.01	0.003
		No	15,737 (98.89)	2162 (98.27)	13,575 (98.99)		
	Depression	Yes	811 (5.10)	169 (7.68)	642 (4.68)	35.28	<0.001
		No	15,102 (94.90)	2031 (92.32)	13,071 (95.32)		
Lifestyle habits and health status	Stress	Feeling an extreme amount	738 (4.63)	110 (5.00)	628 (4.58)	110.97	<0.001
		Feeling a lot	3467 (21.79)	431 (19.59)	3036 (22.14)		
		Feeling a little	9183 (57.71)	1145 (52.05)	8038 (58.62)		
		Hardly feeling	2525 (15.87)	514 (23.36)	2011 (14.66)		
	Smoking	Everyday	2283 (14.35)	428 (19.45)	1855 (13.53)	57.66	<0.001
		Occasionally	387 (2.43)	54 (2.45)	333 (2.43)		
		Smoking in the past but not currently	3721 (23.38)	451 (20.50)	3270 (23.84)		
		Never	9522 (59.84)	1267 (57.60)	8255 (60.20)		
	Drinking	No drinking over the past year	7605 (47.79)	1191 (54.14)	6414 (46.77)	42.31	<0.001
		Once a month	1658 (10.42)	191 (8.68)	1467 (10.70)		
		2–4 times a month	3433 (21.57)	413 (18.77)	3020 (22.02)		
		More than twice a week	3217 (20.22)	405 (18.41)	2812 (20.51)		
	Body mass index			24.16 ± 3.58 [14.76, 42.29]	24.10 ± 3.68 [13.51, 62.64]	0.75	0.450
	Weight management	No management	5193 (32.63)	832 (37.82)	4361 (31.80)	60.37	<0.001
		For loss	6568 (41.28)	783 (35.59)	5785 (42.18)		
		For maintain	3415 (21.46)	444 (20.18)	2971 (21.67)		
		For gain	737 (4.63)	141 (6.41)	596 (4.35)		
	Aerobic physical activity practice	Yes	6785 (42.64)	845 (38.41)	5940 (43.32)	18.67	<0.001
		No	9128 (57.36)	1355 (61.59)	7773 (56.58)		
	Systolic blood pressure			123.2 ± 17.19 [84.0, 211.0]	118.8 ± 16.03 [80.0, 215.0]	11.26	<0.001
	Diastolic blood pressure			76.88 ± 10.08 [40.0, 128.0]	75.21 ± 9.75 [34.0, 129.0]	−1.45	0.150
	Total cholesterol			187.5 ± 39.59 [82.0, 418.0]	192.4 ± 38.43 [66.0, 383.0]	−4.40	<0.001
	Fasting blood glucose			104.5 ± 25.49 [53.0, 335.0]	101.3 ± 22.26 [40.0, 400.0]	5.49	<0.001
	Glycated hemoglobin			5.93 ± 0.88 [4.1, 12.9]	5.79 ± 0.81 [3.1, 15.0]	6.74	<0.001

**Table 2:** Associations Between Household Type and the Prevalence of Hypertension, Dyslipidemia, and Ischemic Heart Disease.

Outcome	Model	OR	95% CI	*p*-Value
Hypertension	Model I	1.818	1.652–2.000	<0.001
	Model II	1.234	1.104–1.378	<0.001
	Model III	1.149	1.022–1.292	0.020
Dyslipidemia	Model I	1.461	1.319–1.618	<0.001
	Model II	1.035	0.925–1.159	0.549
	Model III	1.007	0.893–1.136	0.905
Ischemic heart disease	Model I	1.701	1.368–2.116	<0.001
	Model II	1.169	0.931–1.468	0.179
	Model III	1.143	0.901–1.451	0.270

OR, odds ratio; CI, confidence interval. Multi-person households were used as the reference group. Model I was unadjusted. Model II was adjusted for age and sex. Model III was additionally adjusted for education level, personal income level, smoking, drinking, aerobic physical activity, diabetes mellitus, and depression.

## Data Availability

The data used in this study are publicly available from the Korea Disease Control and Prevention Agency through the Korea National Health and Nutrition Examination Survey data portal, subject to the agency’s data-use procedures.

## References

[1] Gaidai O., Cao Y., Loginov S. (2023). Global cardiovascular diseases death rate prediction. Curr. Probl. Cardiol..

[2] Joseph P., Leong D., McKee M., Anand S.S., Schwalm J.D., Teo K., Mente A., Yusuf S. (2017). Reducing the global burden of cardiovascular disease, part 1: The epidemiology and risk factors. Circ. Res..

[3] Tsao C.W., Aday A.W., Almarzooq Z.I., Anderson C.A.M., Arora P., Avery C.L., Baker-Smith C.M., Beaton A.Z., Boehme A.K., Buxton A.E. (2023). Heart disease and stroke Statistics-2023 update: A report from the American Heart Association. Circulation.

[4] Bhatnagar P., Wickramasinghe K., Wilkins E., Townsend N. (2016). Trends in the epidemiology of cardiovascular disease in the UK. Heart.

[5] Jóźwiak J.J., Studziński K., Tomasik T., Windak A., Mastej M., Catapano A.L., Ray K.K., Mikhailidis D.P., Toth P.P., Howard G. (2020). The prevalence of cardiovascular risk factors and cardiovascular disease among primary care patients in Poland: Results from the LIPIDOGRAM2015 study. Atheroscler. Suppl..

[6] Francula-Zaninovic S., Nola I.A. (2018). Management of measurable variable cardiovascular disease risk factors. Curr. Cardiol. Rev..

[7] Dégano I.R., Marrugat J., Grau M., Salvador-González B., Ramos R., Zamora A., Martí R., Elosua R. (2017). The association between education and cardiovascular disease incidence is mediated by hypertension, diabetes, and body mass index. Sci. Rep..

[8] Ciumărnean L., Milaciu M.V., Negrean V., Orășan O.H., Vesa S.C., Sălăgean O., Iluţ S., Vlaicu S.I. (2021). Cardiovascular risk factors and physical activity for the prevention of cardiovascular diseases in the elderly. Int. J. Environ. Res. Public Health.

[9] Lloyd-Jones D.M., Hong Y., Labarthe D., Mozaffarian D., Appel L.J., Van Horn L., Greenlund K., Daniels S., Nichol G., Tomaselli G.F. (2010). Defining and setting national goals for cardiovascular health promotion and disease reduction: The American Heart Association’s strategic Impact Goal through 2020 and beyond. Circulation.

[10] Gan T., Yang J., Jiang L., Gao Y. (2023). Living alone and cardiovascular outcomes: A meta-analysis of 11 cohort studies. Psychol. Health Med..

[11] Kim H.G. (2019). What are the policy responses to increasing one-person households across the globe?. Glob. Soc. Secur. Rev..

[12] Cohen P.N. (2021). The rise of one-person households. Socius Sociol. Res. A Dyn. World.

[13] Kwon J.S. (2019). Influencing factors on health-related quality of life in middle and old adult one-person households. J. Converg. Cult. Technol..

[14] Schumacher T.L., Burrows T.L., Thompson D.I., Callister R., Spratt N.J., Collins C.E. (2016). The role of family in a dietary risk reduction intervention for cardiovascular disease. Healthcare.

[15] Shim S.B., Lee K.M., Lee K.W., Jeong J.M., Park Y.S., Shin S.Y. (2018). Cardiovascular risk of one-person households. Korean J. Fam. Pract..

[16] Long R.M., Terracciano A., Sutin A.R., Creaven A.M., Gerstorf D., D’Arcy-Bewick S., O’Súilleabháin P.S. (2023). Loneliness, social isolation, and living alone associations with mortality risk in individuals living with cardiovascular disease: A systematic review, meta-analysis, and meta-regression. Psychosom. Med..

[17] Yun K.I., Baek J.U., Choi E.J. (2023). A gender comparative study on risk factors of cardiovascular disease in individuals living alone aged 30–74. Korean J. Health Serv. Manag..

[18] Schultz W.M., Kelli H.M., Lisko J.C., Varghese T., Shen J., Sandesara P., Quyyumi A.A., Taylor H.A., Gulati M., Harold J.G. (2018). Socioeconomic status and cardiovascular outcomes: Challenges and interventions. Circulation.

[19] Zhang Y.B., Chen C., Pan X.F., Guo J., Li Y., Franco O.H., Liu G., Pan A. (2021). Associations of healthy lifestyle and socioeconomic status with mortality and incident cardiovascular disease: Two prospective cohort studies. BMJ.

[20] Farooqi A., Khunti K., Abner S., Gillies C., Morriss R., Seidu S. (2019). Comorbid depression and risk of cardiac events and cardiac mortality in people with diabetes: A systematic review and meta-analysis. Diabetes Res. Clin. Pract..

[21] Malcolm M., Frost H., Cowie J. (2019). Loneliness and social isolation causal association with health-related lifestyle risk in older adults: A systematic review and meta-analysis protocol. Syst. Rev..

[22] O’Súilleabháin P.S., Gallagher S., Steptoe A. (2019). Loneliness, living alone, and all-cause mortality: The role of emotional and social loneliness in the elderly during 19 years of follow-up. Psychosom. Med..

[23] Wadden T.A., Tronieri J.S., Butryn M.L. (2020). Lifestyle modification approaches for the treatment of obesity in adults. Am. Psychol..

[24] Lambrinou E., Hansen T.B., Beulens J.W. (2019). Lifestyle factors, self-management and patient empowerment in diabetes care. Eur. J. Prev. Cardiol..

[25] Gandhi S., Goodman S.G., Greenlaw N., Ford I., McSkimming P., Ferrari R., Jang Y., Alcocer-Gamba M.A., Fox K., Tardif J.C. (2019). Living alone and cardiovascular disease outcomes. Heart.

